# Strategy using a new antigenic test for rapid diagnosis of *Streptococcus pneumoniae* infection in respiratory samples from children consulting at hospital

**DOI:** 10.1186/s12866-020-01764-0

**Published:** 2020-04-07

**Authors:** Cyrille H. Haddar, Johan Joly, Anne Carricajo, Paul O. Verhoeven, Florence Grattard, Olivier Mory, Evelyne Begaud, Yves Germani, Aymeric Cantais, Bruno Pozzetto

**Affiliations:** 1 GIMAP EA 3064 (Groupe Immunité des Muqueuses et Agents Pathogènes), University of Lyon, 42023 Saint-Etienne, France; 2 BioSpeedia, Institut Pasteur, 75015 Paris, France; 3 Laboratory of Infectious Agents and Hygiene, University Hospital of Saint-Etienne, 42055 Saint-Etienne Cedex 02, France; 4 Pediatric Emergency Department, University Hospital of Saint-Etienne, 42055 Saint-Etienne Cedex 02, France

**Keywords:** *Streptococcus pneumoniae*, Respiratory infection, Rapid diagnostic test, PCR assay, Pneumonia, Child

## Abstract

**Background:**

Despite vaccination programs, *Streptococcus pneumoniae* remains among the main microorganisms involved in bacterial pneumonia, notably in terms of severity. The prognosis of pneumococcal infections is conditioned in part by the precocity of the diagnosis. The aim of this study was to evaluate the impact of a Rapid Diagnostic Test (RDT) targeting cell wall polysaccharide of *Streptococcus pneumoniae* and performed directly in respiratory samples, on the strategy of diagnosis of respiratory pneumococcal infections in children.

**Results:**

Upper-respiratory tract samples from 196 children consulting at hospital for respiratory infection were tested for detecting *S. pneumoniae* using a newly-designed RDT (PneumoResp, Biospeedia), a semi-quantitative culture and two PCR assays. If positive on fluidized undiluted specimen, the RDT was repeated on 1:100-diluted sample. The RDT was found highly specific when tested on non-*S. pneumoniae* strains. By comparison to culture and PCR assays, the RDT on undiluted secretions exhibited a sensitivity (Se) and negative predictive value (NPV) of more than 98%. By comparison to criteria of *S. pneumoniae* pneumonia combining typical symptoms, X-ray image, and culture ≥10^7^ CFU/ml, the Se and NPV of RDT on diluted specimens were 100% in both cases.

**Conclusions:**

In case of negative result, the excellent NPV of RDT on undiluted secretions allows excluding *S. pneumoniae* pneumonia. In case of positive result, the excellent sensitivity of RDT on diluted secretions for the diagnosis of *S. pneumoniae* pneumonia allows proposing a suitable antimicrobial treatment at day 0.

## Background

Pneumonia is a major health problem worldwide. It is one of the leading causes of death and morbidity in children, especially in those under 5 years of age [1, 2]. Despite vaccination programs, *Streptococcus pneumoniae* remains among the main microorganisms involved in bacterial pneumonia, notably in terms of severity [3]. The correct management of these infections is helped by a rapid identification of the organism in clinical samples. The microbiological diagnosis of *S. pneumoniae* infection is usually based on the semi-quantitative culture of the bacterium from samples of the respiratory tract (usually sputum or rhino-pharyngeal secretions) [4, 5]. However, several factors may contribute to make the microbiological confirmation difficult: (i) the differentiation between strains of *S. pneumoniae* and of alpha haemolytic streptococci (formerly called viridans) that are part of the commensal oral flora may be difficult [6, 7]; (ii) the correct identification of *S. pneumoniae* compared to other *Streptococcus* species of the mitis group relies usually on the combination of several phenotypic techniques including colony morphology, optochine susceptibility, bile salt lysis and mass spectrometry [6, 8, 9]; (iii) the culture can be falsely negative when the patient has received early antimicrobial treatment. Therefore, the time to get the result of conventional culture, that is usually 18 h, can be delayed, leading to probabilistic antibiotic treatments that impair antibiotic stewardship [10].

In recent years, the search for *S. pneumoniae* urinary antigens has found its place in the bacteriological diagnosis of pneumococcal infections. These tests have a good negative predictive value (NPV) for *S. pneumoniae* pneumonia, but they lack specificity. They are not interesting in paediatrics because of prolonged excretion in case of invasive infections and frequent asymptomatic carriage in this population [11, 12]. Because of these difficulties, it seems necessary to propose new diagnostic approaches for facilitating and shortening the management of pneumococcal infections. The objective of this study was to evaluate the impact of a Rapid Diagnostic Test (RDT) targeting cell wall polysaccharide and performed directly in respiratory samples, on the strategy of diagnosis of respiratory infections potentially caused by this bacterium in children.

## Results

### Analytical performances of the PneumoResp RDT

#### Specificity of the PneumoResp RDT

The specificity of the test was evaluated on 52 bacterial strains belonging to 24 different species, including 30 strains of streptococci of the mitis group (*Streptococcus mitis*, *Streptococcus oralis*, *Streptococcus gordonii*, *Streptococcus sanguinis*, *Streptococcus parasanguinis*, *Streptococcus peroris, Streptococcus pneumoniae* and *Streptococcus pseudopneumoniae*) and 22 strains of bacteria belonging to other genera (*Alloscardovia omnicolens*, *Enterococcus avium*, *Enterococcus faecalis*, *Enterococcus faecium*, *Haemophilus influenzae*, *Haemophilus parainfluenzae*, *Klebsiella pneumoniae*, *Parvimonas micra* and *Staphylococcus aureus*) or to non-mitis streptococci (*Streptococcus agalactiae*, *Streptococcus anginosus*, *Streptococcus constellatus*, *Streptococcus pyogenes*, *Streptococcus salivarius* and *Streptococcus vestibularis*) in order to verify the absence of reactivity with the antigens used in the test with regard to the bacteria potentially present in the oral or respiratory microbiota. No cross-reactions were observed for strains belonging to the tested species, except for one of the 3 strains of *P. micra* and for the 3 strains of *S. pseudopneumoniae*. The PneumoResp RDT was positive for the 4 tested strains of *S. pneumoniae* (3 clinical strains and the ATCC reference strain 49,619) that were used as positive controls.

### Correlation between qPCR assays and semi-quantitative culture

**Fig. 1:**
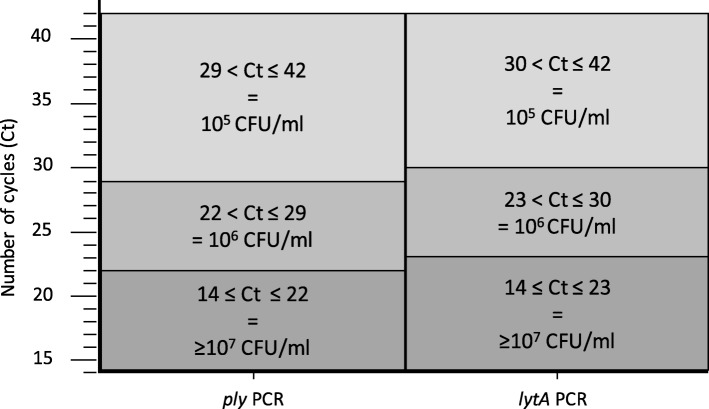
Correlation between qPCR assays and quantitative cultures. The number of cycle threshold (Ct) of two quantitative PCR assays targeting virulence genes of *S. pneumoniae* was correlated to the number of CFU/ml of *S. pneumoniae* by conventional culture

Using dilutions of the ATCC strain 49,619 of *S. pneumoniae*, a correlation was established between bacterial loads obtained by culture (expressed in CFU/ml) and PCR assays (expressed in Ct); the results are shown in Fig. 1. The sensitivity of the two PCR assays was shown to be 10^3^ CFU/ml for the *ply* gene and 10^4^ CFU/ml for the *lytA* gene.

### Clinical performances of the PneumoResp RDT

#### Population and study design

The population consisted of 196 children consulting for symptoms compatible with acute respiratory infection at the University Hospital of Saint-Etienne, France, between October 2017 and July 2018. The study was performed on 196 respiratory samples (sputum or nasopharyngeal secretions) sent to the Microbiology Department of this hospital.

For patients with clinical suspicion of lower respiratory infection, a chest X-ray was performed. The diagnosis of pneumonia was defined on the coexistence of evocative clinical criteria and an abnormal parenchymal image on chest X-ray [3]; *S. pneumoniae* was estimated responsible for pneumonia if the bacterial load was greater than or equal to 10^7^ CFU/ml for *S. pneumoniae* by semi-quantitative culture of respiratory specimens (sputum or nasopharyngeal secretions), irrespectively of the presence of other pathogen(s) [4, 5].

The demographic and clinical characteristics of included patients are listed in Table 1.

**Table 1:** Demographic and clinical characteristics of the studied cohort

Characteristics	Numeric data
Demographic characteristics	
- No. of subjects	196
- mean age in years (range)	1.99 (0–15)
- median age in years	0.83
- interquartile in years	0.18–2.88
- sex ratio M/F	1.23
Main symptom for consulting (%)	
- dyspnea	92 (46.9)
- cough	39 (19.9)
- hyperthermia	77 (39.3)
- other^a^	36 (18.4)
Antibiotic treatment before inclusion (%)	23 (11.7)
Chest radiography (%)	124 (63.3)
- pulmonary condensation	32
- interstitial syndrome	54
- extra-pulmonary abnormal image	17
- no abnormal image	21
Hospital unit at inclusion (%)	
- emergency unit	187 (95.4)
- intensive care unit	9 (4.6)
Hospitalized patients (%)	154 (78.6)
Average length of hospital stay in days	7.8
Biological inflammatory syndrome (%)	63 (31.2)
Pneumonia (%)	86 (43.9)
*S. pneumoniae* pneumonia (%)	23 (11.7)

^a^mainly abdominal pain and diarrhoea

### Overall microbiological results from clinical specimens

Table 2 summarizes the results obtained for the 196 collected respiratory specimens using semi-quantitative culture, qPCR and RDT. All the 70 strains of *S. pneumoniae* that were recovered from culture were susceptible to optochin and positive with the bile salt lysis test.

**Table 2:** Microbiological results concerning the search for *S. pneumoniae* on the 196 samples of the study

Microbiological tests	Number of positive (%)
Semi-quantitative culture	70 (35.7)
- < 10^7^ CFU/ml	20 (10.2)
- ≥ 10^7^ CFU/ml	50 (25.5)
Positive PCR tests (*ply* and/or *lytA)*	169 (86.2)
- *ply* gene	167 (85.2)
• Ct < 22^a^	63 (32.1)
• Ct ≥ 22	104 (53.1)
- *lytA* gene	123 (62.7)
• Ct < 23^a^	32 (16.3)
• Ct ≥ 23	91 (46.4)
Positive PneumoResp RDT	
- undiluted sample	133 (67.8)
- 1:100 diluted sample	76 (38.7)

*Ct* cycle threshold

^a^These thresholds were shown to correspond to approximately 10^7^ CFU/ml by qPCR for the considered PCR assay (see Fig. 1)

### Performances of the RDT by comparison to culture, PCR assays and clinical data

The same 196 respiratory specimens were used to evaluate the performances of the PneumoResp RDT. The RDT was first tested on undiluted fluidized samples; in case of positive result, the test was repeated after a 1:100 dilution. Table 3 shows the performances of the RDT by comparison to the semi-quantitative culture and to the two PCR assays with high bacterial load (Ct corresponding to10^7^ CFU/ml or more); it was also tested with regard to the criteria of *S. pneumoniae* pneumonia that were listed above.

**Table 3:** RDT performances compared to various parameters on 196 and 133 undiluted and 1:100-diluted specimens, respectively

RDT results	*S. pneumoniae* positive culture					
	Positive	Negative	Sensitivity % (IC 95%)	Specificity % (IC 95%)	PPV % (IC 95%)	NPV % (IC 95%)
RDT undiluted			100 (94.8–100)	50.4 (41.4–58.6)	52.6 (48.3–56.9)	100 (94.3–100)
Positive	70	63				
Negative	0	63				
RDT diluted			91.4 (82.5–96)	80.9 (69.6–88.7)	84.2 (76.1–89.9)	89.5 (79.8–94.9)
Positive	64	12				
Negative	6	51				
	*S. pneumoniae* culture ≥10^7^ CFU/ml					
	Positive	Negative	Sensitivity % (IC 95%)	Specificity % (IC 95%)	PPV % (IC 95%)	NPV % (IC 95%)
RDT undiluted			100 (92.9–100)	43.1 (35.4–51.2)	37.6 (34.3–41)	100 (94.3–100)
Positive	50	83				
Negative	0	63				
RDT diluted			94.6 (92.9–100)	68.7 (58.1–77.4)	65.8 (58.3–72.6)	100 (93.7–100)
Positive	50	26				
Negative	0	57				
	Quantitative PCR for *ply* gene Ct < 22					
	Positive	Negative	Sensitivity % (IC 95%)	Specificity % (IC 95%)	PPV % (IC 95%)	NPV % (IC 95%)
RDT undiluted			98.4 (91.5–99.7)	46.6 (38.3–55.1)	46.6 (42.7–50.7)	98.4 (89.8–99.8)
Positive	62	71				
Negative	1	62				
RDT diluted			91.9 (82.5–96.5)	73.2 (61.9–82.1)	75 (67–81.6)	91.2 (82–96)
Positive	57	19				
Negative	5	52				
	Quantitative PCR for *lytA* gene Ct < 23					
	Positive	Negative	Sensitivity % (IC 95%)	Specificity % (IC 95%)	PPV % (IC 95%)	NPV % (IC 95%)
RDT undiluted			100 (87.5–100)	37.3 (30.3–44.8)	20.3 (18.5–22.3)	100 (94.3–100)
Positive	27	106				
Negative	0	63				
RDT diluted			96.3 (81.7–99.3)	52.8 (43.4–62.1)	34.2 (29.6–39.2)	98.3 (89–99.7)
Positive	26	50				
Negative	1	56				
	*S. pneumoniae* pneumonia					
	Positive	Negative	Sensitivity % (IC 95%)	Specificity % (IC 95%)	PPV % (IC 95%)	NPV % (IC 95%)
RDT undiluted			100 (85.7–100)	36.4 (29.6–43.8)	17.3 (15.7–19)	100 (94.3–100)
Positive	23	110				
Negative	0	63				
RDT diluted			100 (85.7–100)	51.8 (42.6–60.9)	30.3 (26.3–34.5)	100 (93.7–100)
Positive	23	53				
Negative	0	57				

*PPV* positive predictive value, *NPV* negative predictive value

**Fig. 2:**
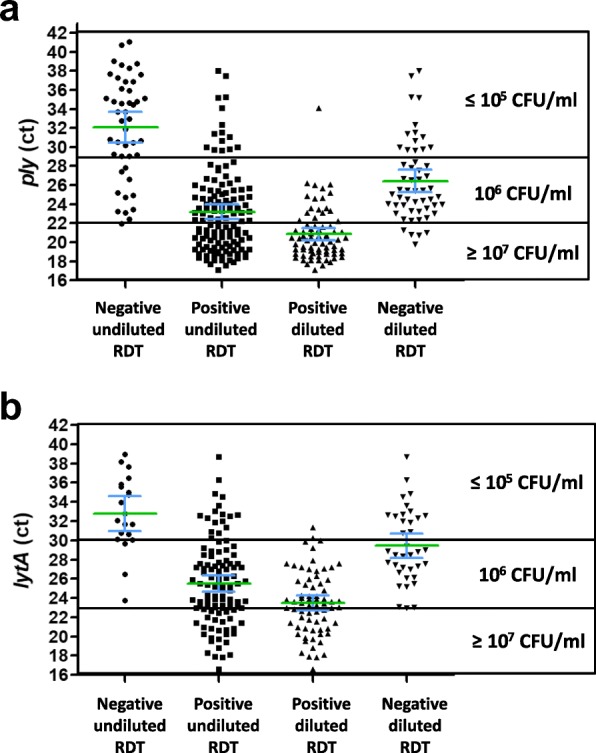
Correlation between qPCR assays and RDT. The approached quantification using *ply* (**a**) or *lytA* (**b**) quantitative PCR assays, as evaluated by cycle threshold (Ct), was correlated to the results obtained with the PneumoResp Rapid Diagnostic Test (RDT) on undiluted and diluted respiratory specimens found either negative or positive. The green bar corresponds to the median and the blue bars to the 95% confidence interval. CFU: colony forming unit

The sensitivity and negative predictive value (NPV) of the RDT on undiluted specimens were shown to be both of 100% by comparison to all the tested conditions depicted in Table 3, except for the *ply* PCR assay, which were both of 98.4. This means that no sample exhibiting a high bacterial load was missed by the RDT and that a negative RDT result was highly predictive of a weak or negative pneumococcal load. Another illustration of the correlation between bacterial loads evaluated by the two PCR assays and the results of RDT is shown on Fig. 2.

**Fig. 3:**
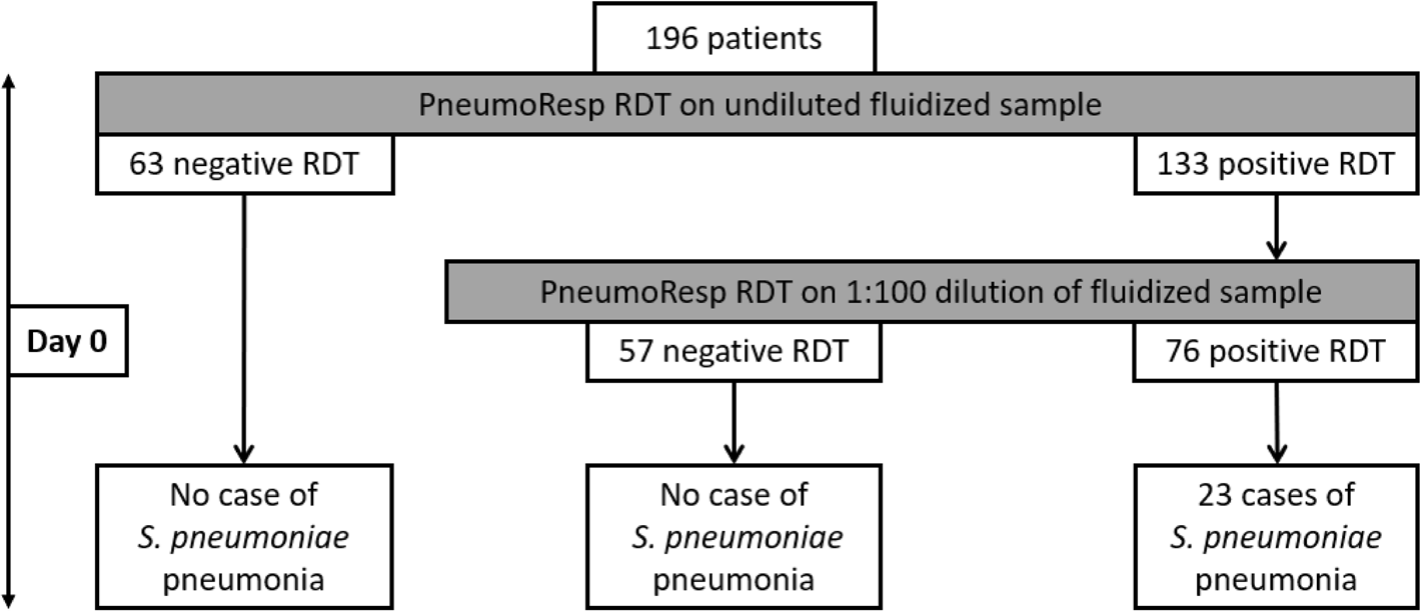
Distribution of pneumococcal pneumonia according to the RDT result at day 0 in the 196 children of the study. A pneumococcal pneumonia was defined by the presence of an abnormal parenchymal image on chest X-ray and a bacterial load of at least 10^7^ CFU/ml in respiratory secretions. RDT: rapid diagnostic test

With regard to the criteria of *S. pneumoniae* pneumonia, the results of Table 3 show that, if the RDT was negative on an undiluted specimen, pneumococcal pneumonia could be ruled out at day 0; in addition, all the 23 presumed pneumococcal pneumonia were tested positive by using the RDT on diluted specimen at day 0 (Fig. 3).

## Discussion

The newly-designed PneumoResp RDT presented in this study was shown able to detect rapidly *S. pneumoniae* antigens with excellent clinical sensitivity in non-invasive respiratory specimens from children suspected of respiratory infection. Moreover, the test was used to give an early measure of the *S. pneumoniae* load in these samples in order to anticipate the treatment of the most serious infections while sparing anti-pneumococcal therapy in case of negative result.

Although the rapid detection of *S. pneumoniae* antigens is currently performed in many body fluids, including urine, cerebrospinal fluid and pleural fluid [3, 7, 11, 12], the specimens from the respiratory tract have been excluded from this clinical practice. A few years ago, a sputum antigen kit was developed in Japan and tested in adult patients for the rapid diagnosis of pneumococcal pneumonia with an excellent sensitivity, exceeding largely that of the urinary tests [13–15]. However, this diagnosis strategy has remained confidential and was never tested in children known to exhibit a high level of pneumococcal asymptomatic carriage [3, 7, 16].

The main strengths of this study can be summarized as follows. First, the present RDT is highly specific for *S. pneumoniae*, notably with regard of the other members of the mitis group. The only cross-reactive results in this group were obtained with *S. pseudopneumoniae*, a species considered to be a respiratory pathogen and having a similar susceptibility profile to antibiotics than *S. pneumoniae* [17–20]. Cross-reaction was also observed with *P. micra*, which was recently reported for another RDT directed against *S. pneumoniae* [21]; this agent is an anaerobic gram-positive bacterium of the oral microbiota that could be responsible for opportunistic infections of the respiratory area. Second, the sensitivity and NPV of the PneumoResp RDT were excellent with regard to semi-quantitative culture and PCR assays targeting virulence pneumococcal genes, which allowed ruling out all the negative results for *S. pneumoniae* at day 0 without risk of missing highly positive samples. Finally, the RDT used on 1:100 diluted samples was able to identify at day 0 all the presumptive cases of pneumococcal pneumonia identified in this study.

This exploratory work has some limitations. First, *S. pneumoniae* is the only microorganism that has been analysed. Although this germ is frequently involved in bacterial pneumonia, other respiratory bacteria such as *H. influenzae* or *Moraxella catarrhalis*, but also “atypical bacteria” (especially *Mycoplasma pneumoniae* and *Chlamydia pneumoniae*) and viruses need to be taken into consideration, notably in children [3, 22–26]. Although not reported in this study, these agents were sought for in all the cases of ascertained pneumonia. Second, it is a retrospective study that was merely dedicated to explore the performances of the test; consequently, the diagnosis strategy that emerged with this two-step RDT would need to be validated prospectively. Third, we considered that a high pneumococcal load in upper respiratory secretions (≥10^7^ CFU/ml or equivalent by PCR) was indicative of an invasive pneumococcal infection; even if this threshold has been recommended in upper respiratory secretions by European guidelines [4, 5], other guidelines discouraged their use [3]. However, the good correlation between quantitative results obtained by culture and PCR (see Fig. 1) indicates that these measures may have a clinical pertinence.

**Fig. 4:**
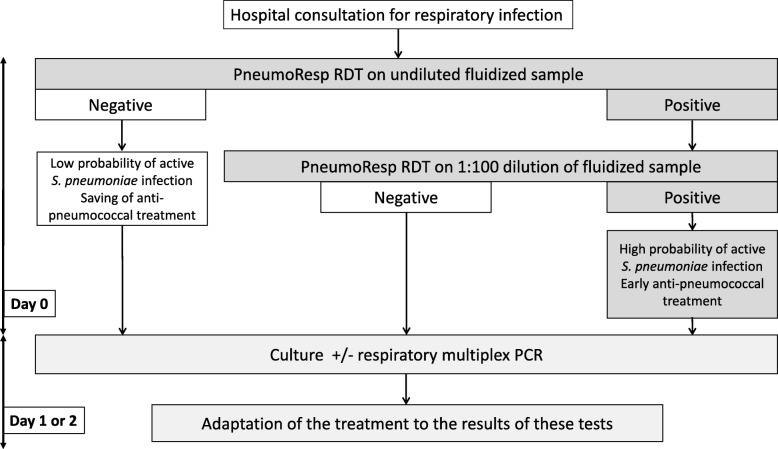
Proposed strategy based on the PneumoResp RDT for orientating the initial (day 0) anti-pneumococcal treatment of children consulting at hospital for respiratory infection. RDT: rapid diagnostic test

The PneumoResp kit, by allowing a two-step analysis, first on undiluted secretions and, if positive, on 1:100-diluted secretions, can be used as a semi-quantification tool. In the conditions of the second step, the sensitivity of the RDT was close to the threshold of 10^7^ CFU/ml considered significant for recognizing the involvement of this bacterium in the aetiology of a pulmonary parenchymal infection [4, 5]. In accordance with the overall results of the study, an algorithm depicted in Fig. 4 summarizes the benefit of the PneumoResp RDT for managing active pneumococcal infections in children. When the RDT is negative on undiluted secretion, the probability of active pneumococcal infection is very low and other aetiology must be looked for. When the RDT is positive on diluted secretion, this probability is high and an anti-pneumococcal treatment must be discussed. In intermediate situations (positive RDT on undiluted secretion and negative on diluted one), the semi-quantitative culture remains essential for deciding between carriage and active infection, as well as the implementation of other tests such as molecular multiplex approaches seeking at the same time the respiratory viruses (the first cause of pneumonia in children) and intracellular atypical bacteria which are difficult to grow and need a different antimicrobial treatment [22–26].

## Conclusions

This pilot study is indicative of the value of the new PneumoResp RDT in children for the early management of active respiratory pneumococcal infection whose incidence is declining with vaccination but whose clinical severity remains high [3, 22, 23]. The strategy described above would help to save useless anti-pneumococcal treatment while identifying rapidly highly active infections. A prospective study on a large cohort of children would be useful to ascertain the present algorithm. Other future research would consists in developing a point-of-care format of this test that would be usable at bedside of emergency units, and in validating this strategy in adult patients whose prevalence of pneumococcal carriage is much more lower than in children.

## Methods

### Microbiological methods

The samples were treated with an equivalent volume of a sterile solution of dithiothreitol (Digest-EUR®, Eurobio) for the realization of semi-quantitative culture. The undiluted pretreated sample and a 1:100 dilution were plated on blood agar (COS, BioMérieux) including an optochin disc (OPTO-F, BioMérieux), Columbia CNA agar (BBL™ Columbia agar, Beckton-Dickinson) and were incubated at 37 °C under 5% CO_2_ atmosphere [17]. The identification of presumptive colonies was based on phenotypic characters (i.e. flat surface and alpha-haemolysis associated or not to optochin susceptibility), then confirmed by Matrix Assisted Laser Desorption Ionization-Time of Flight (MALDI-TOF) (Microflex LT, Bruker Daltonics), after careful analysis of the mass peak profiles, according to the algorithm described by Werno et al. [9]. A positive lysis test using bile salts (BBL Desoxycholate Reagent Droppers, Beckton-Dickinson) was required for validating the MALDI-TOF identification on each isolated strain in order to avoid false-positive results.

### RDT procedure

The newly-designed PneumoResp kit (BioSpeedia) was used. First, 250 μl of sample specimen fluidized by an equal volume of Digest-EUR® was added to tube 1 of the kit and vortex-agitated. One drop of this solution was deposited on the RDT plate, and 3 drops of diluent were immediately added. If no band appeared in the test line and the control band was present within 15 min, the RDT was considered negative and the analysis was stopped. If no band appeared in both marks within 15 min, the RDT was considered invalid. If two bands appeared, the test was considered positive; 10 μl of the sample present in tube 1 and 990 μl of the solution of tube 3 were transferred to tube 2 and vortex-agitated; then, a second RDT was performed on this 1:100 dilution using the same protocol and the same interpretations for the reading. The specificity of the RDT was also tested on 52 bacterial strains (inoculum adjusted to 0.5 McFarland) including 30 streptococci belonging to the mitis group.

### Real-time PCR assays

Two hundred microliters of samples were pretreated with 20 μl of proteinase K (Eurobio) and incubated for 10 min at 50 °C. The samples were then extracted using the EasyMag machine (BioMérieux). Two PCR techniques were used to detect *S. pneumoniae* DNA, one targeting the *ply* gene and the other the *lytA* gene. The primers and probes (Eurogentec) were those used in previous works with minor changes [27–30] (Table 4).

**Table 4:** Sequences of primers and probes used in this study for detection of *S. pneumoniae ply* and *lytA* genes

Primer name	Type	Sequences (5′ to 3′)	Length (bp)	Tm (°C) (GC%)	Reference
***ply***					
SPply F	Forward	TGCAGAGCGTCCTTTGGTCTAT	22	66 (48)	[27]
SPply R	Reverse	CTCTTACTCGTGGTTTCCAACTTGA	25	72 (44)	[27]
SPply TP	Probe	TTCGAGTGTTGCTTATGGGCGCCA	24	74 (54)	[28]
***lytA***					
LytA F	Forward	CGCAATCTAGCAGATGAAGCAG	22	50 (50)	Adapted from [29]
LytA R	Reverse	AAGGGTCAACGTGGTCTGAGT	21	52 (55)	Adapted from [30]
LytA Pr	Probe	TTTGCCGAAAACGCTTGATACAGGG	25	53 (48)	[29]

For amplification of the *ply* gene, the PCR assay was performed under a volume of 20 μl containing 12.5 μl mastermix (1X QuantiTect multiplex PCR kit, Qiagen), 16 pmol each of forward and reverse primers, 6 pmol of probe and 5 μl of DNA template. Thermal cycling reactions consisted of an initial denaturation (10 min at 95 °C) followed by 45 cycles of denaturation (15 s at 95 °C) and annealing/extension (60 s at 60 °C) on the Smartcycler instrument (Cepheid).

For amplification of *lytA* gene, the PCR assay was performed under a volume of 20 μl containing 12.5 μl of mastermix (2X FAST qPCR Mastermix, Eurogentec), 20 pmol each of forward and reverse primers, 6 pmol of probe and 5 μl of DNA template. Thermal cycling reactions consisted of an initial denaturation (10 min at 95 °C) followed by 45 cycles of denaturation (15 s at 95 °C) and annealing/extension (40 s at 59 °C) on the ABI7500 Fast instrument (Applied Biosystems).

### Correlation between bacterial loads obtained by PCR assays and quantitative culture

Using 10-fold serial dilutions of an ATCC reference strain of *S. pneumoniae* (strain 49,619), a correlation was established between the cycle threshold (Ct) of both PCR assays (which is inversely proportional to the DNA bacterial load) and quantitative culture expressed in CFU/ml using a colony counter (Scan 1200, Interscience).

### Statistical analysis

Descriptive variables, sensitivity, specificity and predictive values were reported with their 95% confidence interval (CI) using GraphPad Prism 5 software.

## Data Availability

The datasets used and/or analysed during the current study are available from the corresponding author on reasonable request.

## Abbreviations

ATCC: American type culture collection
CFU: Colony forming unit
Ct: Cycle threshold
NPV: Negative predictive value
PPV: Positive predictive value
qPCR: Quantitative PCR
RDT: Rapid diagnostic test

## References

[1] Bryce J, Boschi-Pinto C, Shibuya K, Black RE (2005). WHO estimates of the causes of death in children. Lancet..

[2] Kassebaum N, Kyu HH, Global Burden of Disease Child and Adolescent Health Collaboration (2017). Child and adolescent health from 1990 to 2015: findings from the global burden of diseases, injuries, and risk factors 2015 Study. JAMA Pediatr.

[3] Harris M, Clark J, Coote N (2011). British Thoracic Society guidelines for the management of community acquired pneumonia in children: update 2011. Thorax..

[4] Freymuth F, Leven M, Wallet F, Cornaglia G, Courcol R, Herrmann JL, Kahlmeter G, Peigue-Lafeuille H, Vila J, SFM, ESCMID (2012). Lower respiratory tract infections. European manual of clinical microbiology.

[5] Botterel F, Cattoen C, Pozzetto B (2018). Infections broncho-pulmonaires. REMIC Société Française de Microbiologie Ed.

[6] Mundy LS, Janoff E, Schwebke KE, Shanholtzer C, Willard KE (1998). Ambiguity in the identification of *Streptococcus pneumoniae*. Optochin, bile solubility, quellung, and the AccuProbe DNA probe tests. Am J Clin Pathol.

[7] Murdoch DR, O’Brien KL, Driscoll AJ (2012). Laboratory methods for determining pneumonia etiology in children. Clin Infect Dis.

[8] Marín M, Cercenado E, Sánchez-Carrillo C, et al. Accurate differentiation of *Streptococcus pneumoniae* from other species within the *Streptococcus mitis* group by peak analysis using MALDI-TOF MS. Front Microbiol. 2017. 10.3389/fmicb.2017.00698. 10.3389/fmicb.2017.00698 PMC5403922 28487677

[9] Werno AM, Christner M, Anderson TP, Murdoch DR (2012). Differentiation of *Streptococcus pneumoniae* from non pneumococcal streptococci of the *Streptococcus mitis* group by matrix-assisted laser desorption ionization-time of flight mass spectrometry. J Clin Microbiol.

[10] Srinivasan A (2017). Antibiotic stewardship: why we must, how we can. Cleve Clin J Med.

[11] Hamer DH, Egas J, Estrella B, MacLeod WB, Griffiths JK, Sempértegui F (2002). Assessment of the Binax NOW *Streptococcus pneumoniae* urinary antigen test in children with nasopharyngeal pneumococcal carriage. Clin Infect Dis.

[12] Viasus D, Calatayud L, McBrown MV, Ardanuy C, Carratalà J (2019). Urinary antigen testing in community-acquired pneumonia in adults: an update. Expert Rev Anti-Infect Ther.

[13] Ehara N, Fukushima K, Kakeya H (2008). A novel method for rapid detection of *Streptococcus pneumoniae* antigen in sputum and its application in adult respiratory tract infections. J Med Microbiol.

[14] Izumikawa K, Akamatsu S, Kageyama A (2009). Evaluation of a rapid immunochromatographic ODK0501 assay for detecting *Streptococcus pneumoniae* antigen in sputum samples from patients with lower respiratory tract infection. Clin Vaccine Immunol.

[15] Ikegame S, Nakano T, Otsuka J (2017). The evaluation of the sputum antigen kit in the diagnosis of pneumococcal pneumonia. Intern Med.

[16] Abdullahi O, Nyiro J, Lewa P, Slack M, Scott JAG (2008). The descriptive epidemiology of *Streptococcus pneumoniae* and *Haemophilus influenzae* nasopharyngeal carriage in children and adults in Kilifi District, Kenya. Pediatr Infect Dis J.

[17] Arbique JC, Poyart C, Trieu-Cuot P (2004). Accuracy of phenotypic and genotypic testing for identification of *Streptococcus pneumoniae* and description of *Streptococcus pseudopneumoniae* sp. nov. J Clin Microbiol.

[18] Laurens C, Michon AL, Marchandin H, Bayette J, Didelot MN, Jean-Pierre H (2012). Clinical and antimicrobial susceptibility data of 140 *Streptococcus pseudopneumoniae* isolates in France. Antimicrob Agents Chemother.

[19] Mohammadi JS, Dhanashree B (2012). *Streptococcus pseudopneumoniae*: an emerging respiratory tract pathogen. Indian J Med Res.

[20] Wen SCH, Anderson T, Murdoch D (2014). Streptococcus pseudopneumoniae. Clin Microbiol Newsl.

[21] Ploton MC, Caseris M, Jost C, et al. Likely false-positive pneumococcal antigen yest BinaxNOW due to *Parvimonas micra*: a four-case series. Chest. 2018. 10.1016/j.chest.2017.12.026. 10.1016/j.chest.2017.12.026 29626971

[22] Bénet T, Sánchez Picot V, Messaoudi M (2017). Microorganisms associated with pneumonia in children <5 years of age in developing and emerging countries: the GABRIEL pneumonia multicenter, prospective, case-control study. Clin Infect Dis.

[23] Leung AKC, Wong AHC, Hon KL (2018). Community-acquired pneumonia in children. Recent Patents Inflamm Allergy Drug Discov.

[24] Cilla G, Oñate E, Perez-Yarza EG, Montes M, Vicente D, Perez-Trallero E (2008). Viruses in community-acquired pneumonia in children aged less than 3 years old: high rate of viral coinfection. J Med Virol.

[25] Esposito S, Daleno C, Prunotto G (2013). Impact of viral infections in children with community-acquired pneumonia: results of a study of 17 respiratory viruses. Influenza Other Respir Viruses.

[26] Cantais A, Mory O, Pillet S (2014). Epidemiology and microbiological investigations of community-acquired pneumonia in children admitted at the emergency department of a university hospital. J Clin Virol.

[27] Corless CE, Guiver M, Borrow R, Edwards-Jones V, Fox AJ, Kaczmarski EB (2001). Simultaneous detection of *Neisseria meningitidis*, *Haemophilus influenzae*, and *Streptococcus pneumoniae* in suspected cases of meningitis and septicemia using real-time PCR. J Clin Microbiol.

[28] Gracie K, Correa E, Mabbott S (2014). Simultaneous detection and quantification of three bacterial meningitis pathogens by SERS. Chem Sci.

[29] McAvin JC, Reilly PA, Roudabush RM (2001). Sensitive and specific method for rapid identification of *Streptococcus pneumoniae* using real-time fluorescence PCR. J Clin Microbiol.

[30] Boving MK, Pedersen LN, Moller JK (2009). Eight-plex PCR and liquid-array detection of bacterial and viral pathogens in cerebrospinal fluid from patients with suspected meningitis. J Clin Microbiol.

